# Microbial signature of plaque and gut in acute coronary syndrome

**DOI:** 10.1038/s41598-023-41867-y

**Published:** 2023-09-07

**Authors:** Eugenia Pisano, Francesca Bugli, Anna Severino, Daniela Pedicino, Francesco Paroni Sterbini, Cecilia Martini, Flavio De Maio, Ramona Vinci, Andrea Sacconi, Francesco Canonico, Alessia D’Aiello, Alice Bonanni, Luca Proto, Pellegrino Ciampi, Myriana Ponzo, Maria Chiara Grimaldi, Andrea Urbani, Aniello Primiano, Jacopo Gervasoni, Rocco Montone, Filippo Crea, Maurizio Sanguinetti, Giovanna Liuzzo

**Affiliations:** 1 Department of Cardiovascular Sciences, Fondazione Policlinico Universitario A. Gemelli IRCCS, Rome, Italy; 2 Department of Cardiovascular and Pulmonary Sciences, Catholic University of the Sacred Heart, Rome, Italy; 3 Department of Laboratory and Infectious Sciences, Fondazione Policlinico Universitario A. Gemelli IRCCS, Rome, Italy; 4 Department of Basic Biotechnological Sciences, Intensivological and Perioperative Clinics, Catholic University of the Sacred Heart, Rome, Italy; 5 UOSD Clinical Trial Center, Biostatistics and Bioinformatics, Regina Elena National Cancer Institute- IRCCS, Rome, Italy

**Keywords:** Cardiology, Medical research

## Abstract

Gut microbiota is an emerging editable cardiovascular risk factor. We aim to investigate gut and coronary plaque microbiota, using fecal samples and angioplasty balloons from patients with acute coronary syndrome (ACS), chronic coronary syndrome (CCS) and control subjects. We examined bacterial communities in gut and coronary plaques by 16S rRNA sequencing and we performed droplet digital PCR analysis to investigate the gut relative abundance of the bacterial genes CutC/CntA involved in trimethylamine N-oxide synthesis. Linear discriminant analysis effect size (LEfSe) at the genus and species levels displayed gut enrichment in *Streptococcus*, *Granulicatella* and *P. distasonis* in ACS compared with CCS and controls; *Roseburia*, *C*. *aerofaciens* and *F. prausnitzii* were more abundant in controls than in patients. Principal component analysis (PCA) of 41 differentially abundant gut taxa showed a clustering of the three groups. In coronary plaque, LEfSe at the genus level revealed an enrichment of *Staphylococcus* and *Streptococcus* in ACS, and *Paracoccus* in CCS, whereas PCA of 15 differentially abundant plaque taxa exhibited clustering of ACS and CCS patients. CutC and CntA genes were more abundant in ACS and CCS than in controls while no significant difference emerged between ACS and CCS. Our results indicate that ACS and CCS exhibit a different gut and plaque microbial signature, suggesting a possible role of these microbiotas in coronary plaque instability.

## Introduction

The pathogenesis of ischemic heart disease (IHD) is complex and results from the interaction between genes and the environment. The genetic background appears to account for less than 20% of cardiovascular disease (CVD) risk, whereas environmental factors account for the remaining 80%^1–3^.

Lifestyle has a major impact on cell physiology, the immune system and metabolism, which is mediated by the resident microbial communities in the intestinal tract. In fact, the gut microbiota operates as an endocrine organ producing multiple compounds that reach the circulation and act on different target organs^4^. A metabolomics approach identified trimethylamine N-oxide (TMAO), derived from gut microbial catabolism of the dietary phosphatidylcholine, choline, betaine and l-carnitine, as an independent risk factor for CVD in large clinical populations^5–8^.

Mechanistically, TMAO accelerates the development of atherosclerosis by promoting cholesterol influx, inhibiting cholesterol efflux, blocking the bile acid pathway and causing excessive activation of platelets^9^.

In addition to the ability of bacterially-derived metabolites to modulate CVD risk, gut microbes can also signal to the host innate immune system through metabolism-independent pathways. In fact, constituents of the bacterial cell wall such as lipopolysaccharide (LPS) and peptidoglycan (PG) can be sensed by host cells through pattern recognition receptors (PRRs), potentiating CVD pathogenesis and progression^10–13^.

The earliest study exploring microbial composition changes associated with carotid atherosclerotic plaques defined a “plaque core microbiota” that partially overlapped with oral and gut intra-individual microbiotas^14^. Patients with stenotic atherosclerotic plaques in the carotid artery harbor exhibit characteristic changes in the gut microbiome, having a greater representation of the genus *Colinsella*, whereas *Roseburia* and *Eubacterium* were prevalent in healthy controls^15^.

Furthermore, the possible involvement of the gut microbiome in coronary plaque instability has been hypothesized by the evidence of a different gut microbial composition, and in particular an increased abundance of *Streptococcus*, in relation to the severity of coronary artery disease (CAD)^16, 17^. Increased gut microbial translocation into systemic circulation after ST-elevation myocardial infarction (STEMI) leads to enhanced inflammation and has a considerable prognostic value for cardiovascular outcome^18^.

The present study is the first to compare gut and coronary plaque microbiotas in patients with chronic coronary syndrome (CCS) and in patients with ACS, both with non-ST elevation myocardial infarction (NSTEMI) and STEMI. To this end, the profiling of gut and coronary plaque metagenomes, based on DNA obtained from stool samples and plaque material rescued from angioplasty balloons used during percutaneous coronary intervention (PCI), was performed. In addition, an analysis of bacterial genes encoding choline TMA-lyase (CutC) and Rieske-type oxygenase (CntA), which are associated with TMA synthesis from choline and carnitine, respectively, was conducted on the stool of patients and controls.

## Methods

### Study populations

We enrolled patients admitted to our hospital with (1) a diagnosis of ACS (n = 44), of which 29 with NSTEMI and 15 with STEMI); (2) CCS (n = 28), with symptoms of stable effort angina lasting more than 12 months and no angina episodes during the previous 48 h, and (3) controls (n = 34) without overt history of ischemic heart disease. The controls, recruited at our cardiovascular primary prevention outpatient clinic, did not differ from patients for sex, body mass index and diabetes incidence. The diagnosis of CCS, NSTEMI, and STEMI was made based on current guidelines^19–21^. All individuals gave their informed consent. The Ethics Committee of the Fondazione Policlinico A. Gemelli, IRCCS-Catholic University of Sacred Heart of Rome approved the study (for details on exclusion criteria see Supplemental Material). All methods were performed in accordance with the relevant guidelines and regulations.

### Angioplasty balloon and stool sample collection

See Supplemental Material.

### Bacterial DNA extraction

See Supplemental Material.

### Evaluation of the presence of bacterial rDNA 16S in balloon samples by qualitative PCR

See Supplemental Material.

### 16S rDNA sequencing

The 16S rRNA gene V3–V4 region was amplified using degenerate forward Pro341F and reverse Pro805R primers^22^. The DNA library was obtained as described by the MiSeq rRNA amplicon sequencing protocol^23^, and the sequencing reactions were performed on an Illumina MiSeq platform (San Diego, CA) using the MiSeq reagent kit version 3.

### Sequence data analysis

We used a combination of the software packages QIIME (v1.9.1)^24^ and VSEARCH (v1.1)^25^ to perform the sequence data analysis. FASTQ raw paired-end Read 1 and Read 2 were merged using the FASTQ join option, followed by a quality-filtering step at the Q20 level in QIIME. VSEARCH’s global pairwise sequence comparison function was used to perform all-vs.-all alignment, clustering, chimera detection and searches. Briefly, sequences were clustered into Operational Taxonomic Unit (OTUs) at 97% similarity after chimeric sequence removal. To determine the taxonomy of representative sequences from each OTU, we used the UCLUST consensus taxonomy classifier^26^ using queries against the Greengenes taxonomy reference database (v13.8.0) (sequence identity threshold of 97%). A biological observation matrix (BIOM)^27^ was generated at different taxonomic levels (from phylum to species).

### Design of degenerate primers targeting the CutC and CntA genes

See Supplemental Material.

### Droplet digital PCR (ddPCR) of CutC and CntA genes

ddPCR (QX200 Droplet Digital PCR System, Bio-Rad Laboratories, Hercules, USA) was used to assess differences in CutC and CntA gene abundance between CCS, ACS and controls. We employed 16S rRNA abundances for normalization (for details see Supplemental Material).

### Quantification of serum trimethylamine N-oxide levels

See Supplemental Material.

### Statistical analysis

To analyze the resulting BIOM table, MicrobiomeAnalyst (http://www.microbiomeanalyst.ca), was used^28^. First, data were filtered to remove low-quality features and then normalized by rarefaction to the minimum library size^29^. Low abundance features (less than 4 counts and a prevalence in samples under 20%) and low variance features (interquartile range less than 10%) were removed. Total sum scaling (TSS) normalization was performed.

The β-diversity (between-sample diversity) was estimated by calculating the dissimilarity matrix using phylogenetic-based distance metrics (unweighted UniFrac). Principal coordinate analysis (PCoA) 2D ordination plots were generated to visualize the dissimilarity matrices. The statistical significance of sample groupings was assessed by PERMANOVA. Taxonomic analysis was performed using the LEfSe method, which compares the 16S abundance profiles between samples in different states^30^.

Significant features at the OTU level were selected using the Kruskal–Wallis test and linear discriminant analysis (LDA). OTUs with p values less than 0.05 and LDA scores higher than 2 were considered statistically significant. Additionally, a Wilcoxon rank-sum test was evaluated for each comparison. The Benjamin Hockeberg False Discovery Rate procedure was applied.

A Spearman rank correlation was used to investigate the association of selected features with clinical variables, risk factors and therapies.

A functional analysis was performed with the PICRUSt algorithm to build the Kyoto Encyclopedia of Genes and Genomes (KEGG) Orthologies (KO) and Cluster of Orthologous Group (COG) matrix of abundance. COG and KO were analyzed by the SHOTGUN procedure to assess enriched pathways.

## Results

### Characteristics of the study participants

A total of 72 consecutive patients were enrolled in the intensive and subintensive cardiovascular care unit of IRCCS Fondazione Policlinico A. Gemelli and were divided into the following groups: (1) 44 patients had a diagnosis of ACS (29 NSTEMI [66%] and 15 STEMI [34%], age 65 ± 12 years, 33 males); (2) 28 patients (39%) had a diagnosis of CCS (age 67 ± 9 years, 23 males); and (3) 34 subjects (age 58 ± 11, 23 males) were controls.

Table 1 summarizes the demographic data, the cardiovascular risk factors, the therapies and the clinical data in the ACS, CCS, and control groups.

**Table 1:** Demographic and clinical characteristics of the study population.

	ACS (n = 44)	CCS (n = 28)	Controls (n = 34)	p-value
Sex, male/female	33/11	23/5	23/13	0.2
Age, years	65 ± 12	67 ± 9	58 ± 11	0.01*^†^
BMI, kg/m^2^	28 ± 5	27 ± 3.4	30 ± 8	0.06
Risk factors				
Hypertension	33 (75)	24 (86)	19 (56)	0.028^†^
Dyslipidemia	29 (66)	26 (93)	13 (38)	< 0.0001*^†‡^
Obesity (BMI > 30)	15 (34)	5 (18)	13 (38)	ns
Current smoke	13 (29)	3 (11)	3 (9)	0.03*
Family history of IHD	15 (34)	13 (46)	8 (23)	ns
Diabetes	15 (34)	12 (43)	12 (35)	ns
Previous ACS	7 (16)	19 (36)	–	ns
Pharmacological treatment at enrollment				
ASA	13 (29)	23 (82)	8 (23)	< 0.0001^†‡^
P2Y12 inhibitors	6 (14)	11 (39)	–	0.02
Anticoagulants	3 (7)	2 (7)	2 (6)	ns
Beta-blockers	16 (36)	20 (71)	7 (20)	0.0002^†‡^
Diuretics	9 (20)	6 (21)	5 (21)	ns
ACEi	21 (48)	9 (32)	9 (36)	ns
ARBs	6 (14)	9 (32)	5 (14)	ns
Statins	18 (41)	20 (71)	4 (12)	< 0.0001*^†‡^
Ca-antagonists	9 (20)	7 (25)	3 (9)	ns
Insulin	4 (9)	3 (11)	3 (9)	ns
OHA	10 (23)	11 (39)	11 (32)	ns
PPIs	13 (29)	16 (57)	6 (18)	0.0036^†‡^
Laboratory assays				
TC, mg/dL	183 (139–203)	160 (111–169)	175 (142–210)	0.02^‡^
LDL-C, mg/dL	110 ± 36	83 ± 30	106 ± 34	0.03^‡^
HDL-C, mg/dL	40 ± 13	45 ± 12	51 ± 15.3	0.009*
TG, mg/dL	112 (96–159)	98 (76–138)	112 (87–161)	ns
cTNT > 0.004 ng/mL	31(75)	7 (25)	–	0.0002
Hs-CRP, mg/L	12 (4.4–30)	0.8 (0.5–2.6)	0.5 (0.5–2.4)	< 0.0001*^‡^
Creatinine, mg/dL	0.91 (0.78–1.13)	0.88 (0.77–1.21)	0.8 (0.73–0.94)	ns
Hemoglobin, g/dL	14.1 ± 1.9	14 ± 1.4	13.9 ± 1.2	ns
Platelets, 10^3^/mL	246 ± 68.1	220 ± 35.3	245 ± 57.6	ns
Neutrophil count, 10^9^/L	7.6 ± 3	4.8 ± 1.6	4.6 ± 1.6	< 0.0001*^‡^
Lymphocyte count, 10^9^/L	1.9 ± 1	2 ± 0.6	2.2 ± 0.5	ns
Monocyte count, 10^9^/L	0.5 ± 0.2	0.5 ± 0.2	0.4 ± 0.1	0.009*

The clinical and demographic characteristics of the ACS and CCS patients and controls enrolled in the study are shown. Values are expressed as mean ± SD, n, n (%), or median (IQR).

*ACS* acute coronary syndrome, *ACEi* angiotensin converting enzyme inhibitors, *ARBs* angiotensin receptor blockers, *ASA* acetylsalicylic acid, *BMI* body mass index, *CCS* chronic coronary syndromes, *HDL-C* high density lipoprotein cholesterol, *IHD* ischemic heart disease, *LDL-C* low density lipoprotein cholesterol, *OHA* oral hypoglycaemic agents, *PPIs* proton pomp inhibitors, *TC* total cholesterol, *TG* triglycerides, *SD* standard deviation, *IQR* interquartile range.

*p < 0.05 between ACS patients and controls; ^†^p < 0.05 between CCS patients and controls; ^‡^p < 0.05 between ACS and CCS patients.

In summary, ACS patients, compared with CCS patients, showed significantly higher levels of granulocytes, total and LDL-cholesterol, and high sensitive-C-Reactive Protein (hs-CRP). Regarding therapy, ACS, CCS and control subjects were different for aspirin, P2Y12 receptor inhibitors, statins, β-blockers and proton pomp inhibitors (PPI).

### Taxonomic profile of the gut microbiota in ACS and CCS, and control groups

A richness analysis was performed in the gut microbiome of the patients with ACS, CCS and controls using the Shannon index to display the alpha diversity. No significant difference was found (Fig. S1).

To assess difference in the overall composition (β-diversity) of the gut microbiota, a PCoA was constructed with the three groups (F: 1.49; R^2^: 0.03; p < 0.02) (Fig. S2a). The analysis revealed significant differences in microbial composition between ACS and controls and (F: 1.66, R^2^: 0.02; p < 0.03), CCS and controls (F: 1.75, R^2^: 0.03; p < 0.02), whereas the comparison between ACS and CCS did not show a significant difference (Fig. S2b).

**Figure 1:**
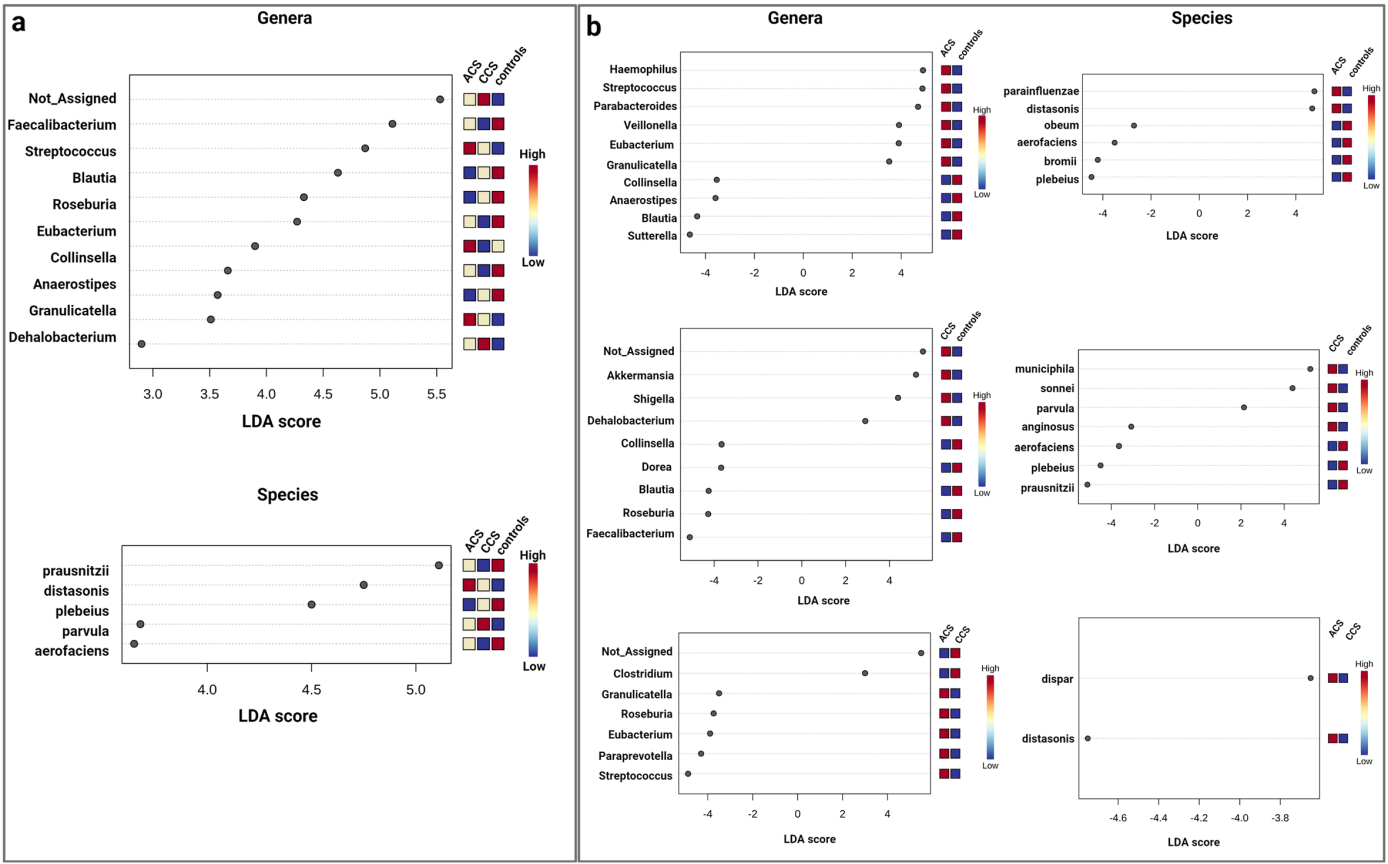
(**a**) Linear discriminant analysis (LDA) effect size (LEfSe) analysis among ACS, CCS and controls in the gut. LEfSe analysis detects statistically significant changes at the genus and species levels among the three groups. (**b**) LEfSe analysis detects statistically significant changes at the genus and species levels in ACS vs. controls, controls vs. CCS and ACS vs. CCS. Microbial taxa that are different between groups are highlighted with log differences on the x-axis (p < 0.05).

The LEfSe analysis at the genus level showed an enrichment of *Streptococcus* (p = 0.01) and *Granulicatella* (p = 0.04) in ACS compared with CCS patients and controls while revealing an enrichment of some butyrate-producing bacteria, including *Faecalibacterium* (p = 0.05) and *Roseburia* (p = 0.02), in controls compared with patients (Fig. 1a).

At the species level, the LEfSe analysis showed an enrichment of *Parabacteroidetes distasonis* (p = 0.005) in ACS compared with CCS patients and controls, whereas *Collinsella aerofaciens* (p = 0.002) and *Faecalibacterium prausnitzii* (p = 0.05) were more abundant in controls than in patients (Fig. 1a). In addition, enrichment of the *Haemophilus* (p = 0.02) and *Clostridium* genera (p = 0.04) was evident in ACS vs. controls and in ACS vs. CCS, respectively (Fig. 1b).

**Figure 2:**
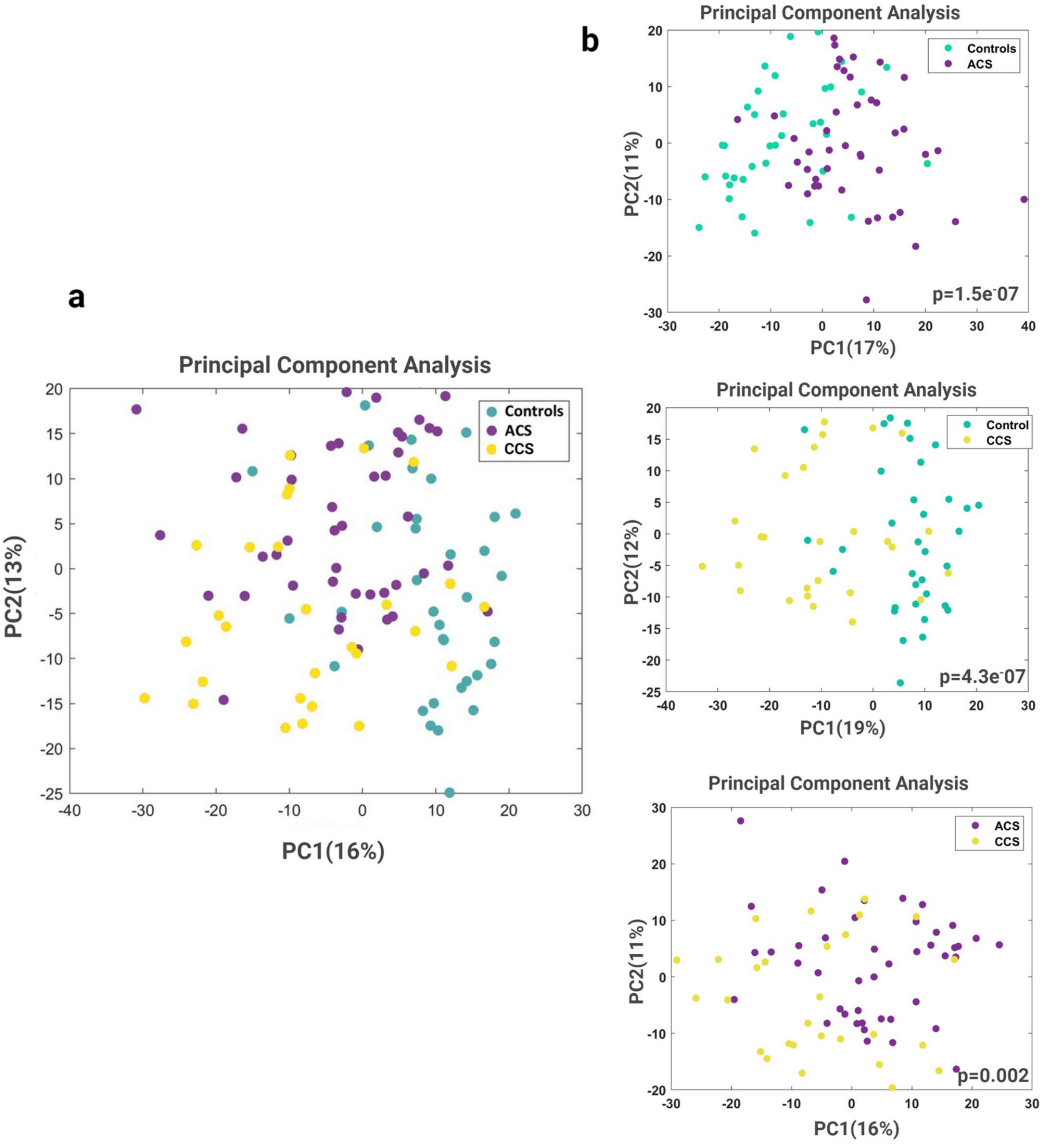
Principal component analysis (PCA) based on deregulated OTUs in the gut. (**a**) PCA between controls, ACS patients and CCS patients. (**b**) CCS vs. controls, p = 4.3e−07; ACS vs. controls, p = 1.5e−07 and ACS vs. CCS, p = 0.002. Wilcoxon test was used.

A significant clustering of ACS, CCS and controls was evident in the PCA of 41 selected OTUs identified by LEfSe (Fig. 2a). The individual comparisons are represented in Fig. 2b: CCS vs. controls, p = 4.3e−07; ACS vs. controls, p = 1.5e−07 and ACS vs. CCS, p = 0.002. To explore the relationship between the clinical features, risk factors and the relative abundance of deregulated taxa in the three groups, we performed a Spearman correlation analysis. Only significantly different clinical parameters, in the three groups, were analyzed. Clinical features linked, both to the inflammatory burden (hs-CRP) and to the innate immune response (monocyte/neutrophil count), correlated positively with *Streptococcus*. Among markers associated to lipid metabolism, TC and LDL-C correlated with several bacterial taxa including *Clostridiales, Lachnospiraceae, Blautia, Collinsella* and *Coprococcus*, some of which have already been associated with serum lipoproteins (Fig. S3a)^14, 31^.

Regarding the CVD risk factors, smoking habit correlated positively with *Enterobacteriaceae* and negatively with *Collinsella* and *Blautia*, dyslipidemia correlated with *Bacteroides*, *Parabacteroides* and *Dehalobacterium*, while hypertension correlated positively only with *Dehalobacterium* (Fig. S3b).

Concerning the influence of pharmacological therapies, we analyzed the correlation between aspirin (ASA), P2Y12 inhibitors, beta-blockers, statins, PPI and the deregulated bacterial taxa (Fig. S3c). Anti-platelets therapies (ASA and P2Y12), statins and PPI were associated with a lower abundance of *Roseburia* and *Blautia*; *Dorea* and *Prevotella* were also down-represented in patients in therapy with ASA*.* Statins were positively correlated with *Parabacteroides*. Finally, beta-blockers were positively correlated with *Streptococcus*.

### Gut microbiome predicted functional capacity analysis in ACS, CCS, and control groups

**Figure 3:**
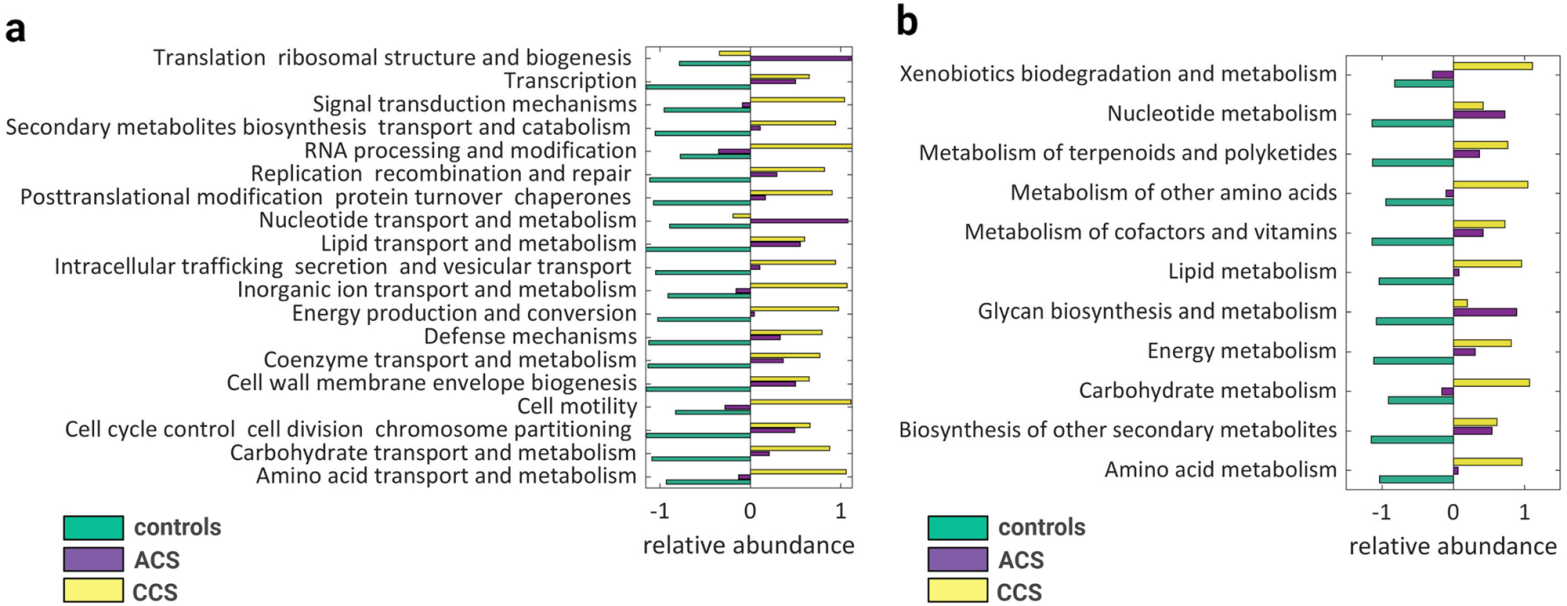
Changes in the functional potential of gut microbiota in ACS, CCS and controls. (**a**) Differential PICRUSt-predicted functional categories based on the COG database in ACS, CCS and controls; (**b**) differential PICRUSt-predicted KEGG pathways between ACS, CCS and controls.

To improve the insight into functional changes within the ACS and CCS gut microbiomes compared with controls, we used PICRUSt to infer metagenome functional content based on the microbial community profiles. As depicted in Fig. 3, the microbial communities present in the three groups could be distinguished based on their predicted function. The microbiome from ACS and CCS patients showed an enriched potential for bacterial replication metabolism-related pathways based on COG database and analysis (Fig. 3a). The difference can be appreciated even between ACS and CCS, suggesting a role of bacterial proliferation in ACS. The predicted KEGG pathways that significantly differ between ACS and CCS can be annotated in the functional capacity of glycan biosynthesis, such as LPS and PG, which were clearly more represented in ACS patients. Of note, significant differences were also observed in pathways related to xenobiotic biodegradation and metabolism, which were enriched in CCS patients (Fig. 3b).

### Taxonomic profile of the plaque microbiota in ACS and CCS

**Figure 4:**
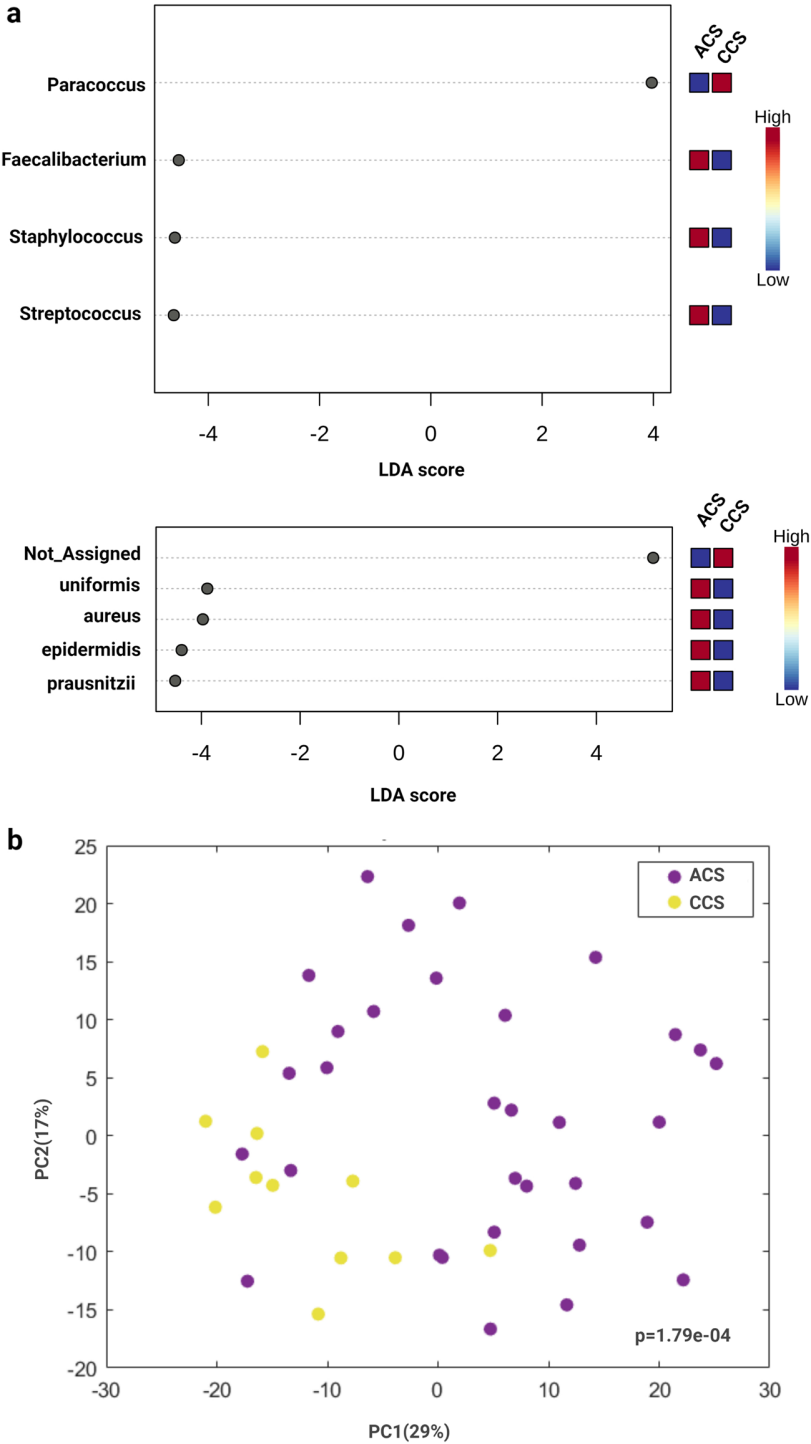
Linear discriminant analysis (LDA) effect size (LEfSe) analysis between ACS and CCS in coronary plaques. (**a**) The LEfSe analysis detects statistically significant changes at the genus and species levels between the two groups. Microbial taxa that are different between groups are highlighted with log differences on the x-axis (p < 0.05). (**b**) Principal component analysis (PCA) based on deregulated OTUs in coronary plaques.

The coronary plaque microbiota was evaluated by sequencing of DNA extracted from angioplasty balloon-derived plaque material in patients with ACS and CCS undergoing PCI. To assess whether relevant differences in the bacterial taxonomic composition of coronary plaque were present between the two groups, a PCoA (β-diversity) was constructed. The analysis revealed no significant differences in the microbial composition between ACS and CCS patients (Fig. S4). However, the LEfSe analysis at the genus level showed a significant enrichment of *Faecalibacterium*, *Staphylococcus*, and *Streptococcus* in ACS compared with CCS (p < 0.05), and an enrichment of *Paracoccus* in CCS compared with ACS (p < 0.05). Interestingly, *Streptococcus* emerged as a relevant genus both in the gut and in coronary plaques in ACS patients. At the species level, the LEfSe analysis showed an enrichment of *Bacteroides uniformis*, *Staphylococcus aureus*, *Streptococcus epidermidis* and *Faecalibacterium prausnitzii* in ACS patients (Fig. 4a).

A significant (p = 1.79e−04) clustering of ACS and CCS patients was evident in the PCA of 15 selected OTUs identified by LefSe (Fig. 4b).

### Overall comparison of gut and coronary plaque microbiotas

An overall comparison of gut and coronary plaque microbiotas was performed. Alpha and β-diversity showed a difference in bacterial richness and composition, respectively, in the two body habitats (α-diversity, p = 1.2192e−16; β-diversity, F: 79.102; R^2^: 0.41177; p = 0.001) (Fig. S5). A comparison of the bacterial composition at the phylum and genus levels (using the 20 most abundant genera) is represented as the mean abundance in Fig. S6. The gut microbiota showed a heterogeneous composition with a pronounced presence of *Bacteroidetes* and *Firmicutes*, while the coronary plaques were enriched in microbes with proinflammatory phenotypes belonging mainly to *Proteobacteria*. This is expected given the anaerobic nature of the gut, while the plaque is directly exposed to blood oxygen.

The most relevant genera observed in the coronary plaque of both groups were *Ralstonia*, *Burkholderia*, *Propionibacterium*, *Streptococcus* and *Staphylococcus.*

### Abundance of CutC and CntA genes in gut and TMAO serum level

**Figure 5:**
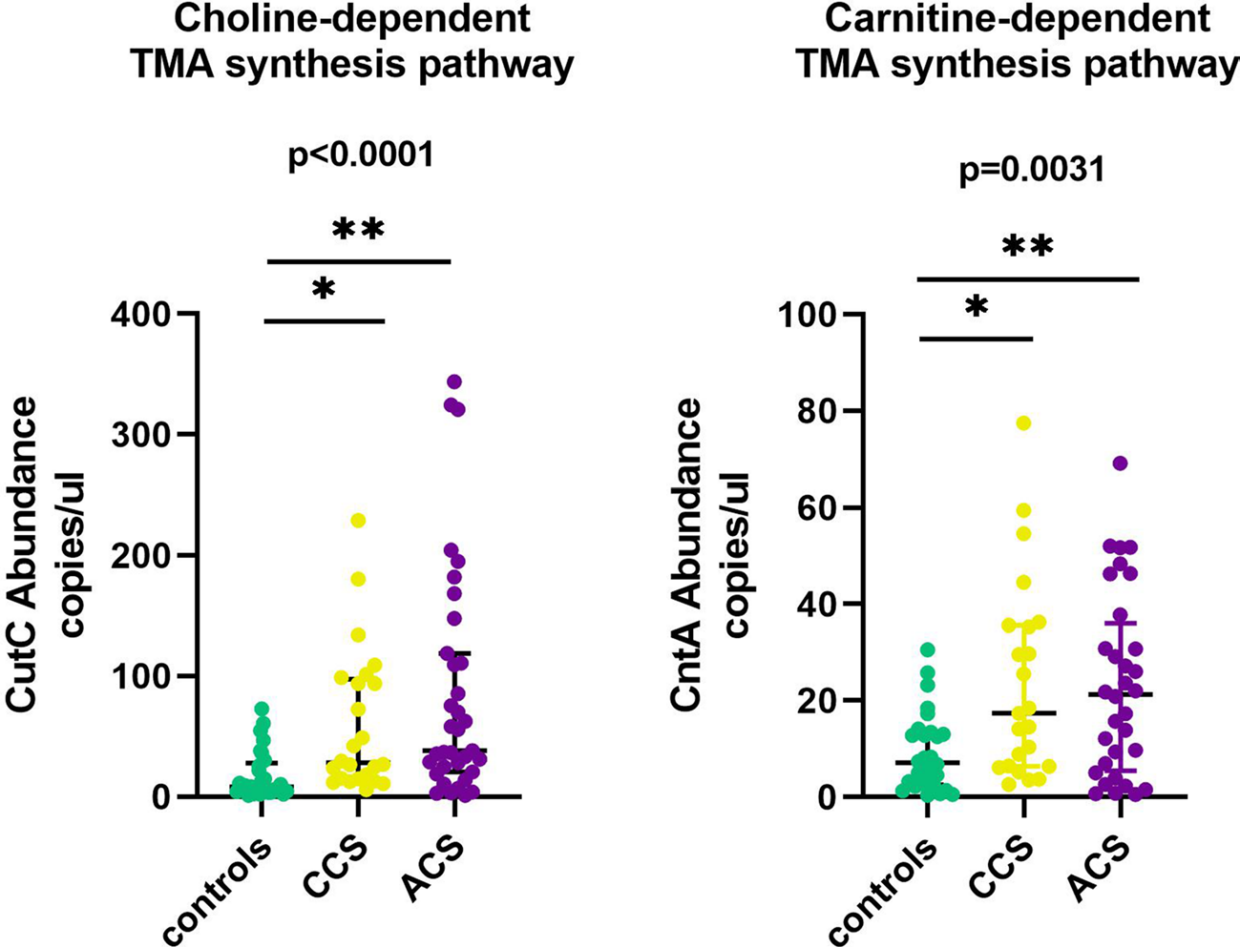
Abundance analysis of choline TMA-lyase gene CutC and Rieske-type oxygenase gene CntA in the gut of ACS, CCS and controls. Choline-dependent TMA synthesis pathway: CutC gene is significantly more abundant in ACS and CCS patients compared with controls, p = 0.0001 and p = 0.0012, respectively; Carnitine-dependent TMA synthesis pathway: CntA gene is significantly more abundant in ACS and CCS patients compared with controls, p = 0.013 and p = 0.0074, respectively.

The microbiome-derived metabolite TMA is produced from choline and carnitine through two major TMA-synthesis pathways involving a specific glycyl radical enzyme, choline TMA-lyase (CutC) and its activator CutD, and a two component Rieske-type oxygenase/reductase (CntA/B)^32, 33^. Using ddPCR, we quantified the CutC and CntA genes in the metagenomes of ACS, CCS, and controls. We found an increased abundance of CutC in ACS and CCS patients compared with controls (ANOVA p < 0.0001; ACS vs control, p = 0.0001; CCS vs controls, p = 0.0012) and CntA (ANOVA p = 0.0031; ACS vs. controls, p = 0.013; CCS vs. controls, p = 0.0074) (Fig. 5).

In particular, among patients with ACS, those with STEMI showed a higher abundance of CutC and CntA (Fig. S7). Analysis of TMAO serum levels revealed no significant difference between ACS and CCS patients. Although not statistically significant, patients with STEMI showed an increased level of TMAO compared with NSTEMI and CCS (Fig. S8).

## Discussion

To the best of our knowledge, this is the first study to conduct a metagenomics analysis in the gut of ACS and CCS patients and controls, also associating a comparison of coronary plaques from ACS and CCS, to highlight the contribution of both microbiotas to coronary instability. This metagenomics analysis reveals that the taxonomic composition of gut microbiota is significantly different between controls and CAD patients, whereas the comparison between ACS and CCS patients does not show a significant diversity. A deeper exploration indicates a higher abundance of the *Streptococcus* and *Granulicatella* in ACS than in CCS patients and controls. In contrast, an enrichment of some butyrate-producing bacteria such as *Faecalibacterium* and *Roseburia*, which are associated with the well-being of the gut microbiota, has been found in controls compared with patients with CAD^34^.

The functional capacities revealed a remarkable difference between ACS, CCS, and controls in several bacterial metabolic pathways. ACS exhibited a higher potential for both bacterial replication and glycan biosynthesis than CCS patients, whereas xenobiotic biodegradation and metabolism pathways were more represented in CCS patients who had received chronic preventive treatments more frequently than ACS.

Emerging data support a direct drug effect on microbiome composition both as single and combinatorial medications (polypharmacy). For example, an increased abundance of oral based strains *Rothia*, *Haemophilus* and *Streptococcus* species was observed in the gut of individuals taking proton pomp inhibitors, while the combination of beta-blockers with diuretic agents leads to an enrichment of intestinal *Roseburia*. Overall, several medications for cardiometabolic disease lead to a microbiome shift that might mediate the improvement in clinical markers^35^.

Our analysis, while not highlighting the same associations, probably due to the small sample size, suggests that the taxa dysregulation observed in the three groups was more related to the clinical phenotype than to pharmacological therapy.

The comparison between gut and plaque microbiotas revealed a different microbial profile between the two sites. Whereas the gut microbiota showed a more heterogeneous composition with a pronounced presence of *Bacteroidetes* and *Firmicutes*, the plaques were enriched in microbes with proinflammatory phenotypes belonging mainly to *Proteobacteria*. These results are in line with data on carotid plaque microbial composition^14^.

The genera that we found in coronary plaques are involved in skin and respiratory infections or come from the buccal mucosa, suggesting that the bacteria found in coronary plaques could move from body sites other than the gut. These observations suggest a selective retention of proinflammatory bacteria, sustained by the microenvironment of the atherosclerotic plaques, forming a unique core microbiota, as previously identified in carotid atherosclerotic plaques^14, 36^ independent from clinical diagnosis. Once again, the relative abundance of *Streptococcus,* was higher in ACS relative to CCS*.*

Experimental mouse models of atherosclerosis, colonized with synthetic microbial communities producing butyrate, showed an improvement in atherosclerotic burden and a reduction in systemic inflammation suggesting that variation in the levels of these bacteria is causally linked to CVD development^37^. Conversely, the species belonging to bacterial genera found in the coronary plaques are able to trigger the release of the neutrophil extracellular traps (NETs), favoring immuno-thrombosis, and to accelerate the atherosclerosis progression.

Indeed, ACS patients presenting with markedly increased gut permeability, showed significantly enhanced LPS at systemic (serum) and local (thrombus) level, thus providing a further evidence of the systemic inflammatory burden in patients with plaque instability^38–40^.

Infectious triggers have been proposed in the past, as additional risk factors of ACS^41, 42^ even if it has never been convincingly shown, as witnessed by the failure of antibiotic treatment against *Chlamydia pneumoniae* in improving the outcome of ACS.

The results of our study support the notion that, microbial triggers might play a direct and/or indirect role in plaque destabilization. Indeed, the differences found in the gut microbiota between ACS and CCS patients reflect a different metabolome that might impact disease in the coronary circulation^43^. The presence of proinflammatory bacteria in the coronary plaques could also induce a local inflammatory response through LPS or PG signaling, as previously demonstrated in the adipose tissue surrounding epicardial coronary arteries, in which innate immunity was activated in ACS patients harboring a local proinflammatory bacterial signature^44^.

The growing knowledge of the role of the bacteria-derived metabolite TMAO in cardiovascular risk prompted us to investigate the bacterial genes related to TMA production from the choline and carnitine catalytic pathways. Patients with ACS and CCS showed an increased abundance of CutC and CntA genes relative to controls; in particular, patients with STEMI had the highest level of TMA-related genes. Although the quantification of serum levels of TMAO showed the same trend, we failed to obtain a significant difference between groups, and we failed to demonstrate a correlation between TMAO levels and CutC/CntA gene abundance. Possible explanations for this apparent discrepancy might be that TMAO serum levels depend not only on TMA bacterial synthesis but also on its oxidation by hepatic flavin monooxygenases and on its urinary clearance.

In conclusion, our study shows not only that the gut microbiome is different between patients with CAD and controls but also, more importantly, that there are some differences between patients with ACS and CCS, suggesting an active role of gut microbes in coronary instability. Where this is the case, it might open the way to new forms of treatment for the prevention of ACS by intervention that modify the gut microbiota. Furthermore, our work suggests the presence of a microbiota in coronary plaque, in which the local environment selectively retains proinflammatory bacteria.

### Study limitations

The major limitation of the study is the associative nature of the data that does not prove a specific pathogenic role of the microbiome in coronary instability. However, these findings open the way both to longitudinal studies that will define whether the microbiome pattern we found in ACS is specific to the acute phase of the disease and to mechanistic studies with animal models of atherosclerosis to establish the molecular mechanisms triggered by microbiota. Another limitation is the small number of patients included in the study. Although recent studies on microbiota analyses in chronic diseases are supported by larger cohorts, our population study included acute patients with NSTEMI and STEMI in which the collection of stool samples experienced difficulties. However, to the best of our knowledge, this is the study that compares the largest number of acute versus chronic patients in CAD.

### Supplementary Information

Supplementary Information.

## Data Availability

The datasets generated and/or analyzed during the current study are not publicly available but are available from the corresponding author on reasonable request.

## Abbreviations

CVD: Cardiovascular disease
CAD: Coronary artery disease
ACS: Acute coronary syndrome
CCS: Chronic coronary syndrome
LEfSe: Linear discriminant analysis effect size
OTU: Operational taxonomic unit
PCA: Principal component analysis
PCI: Percutaneous coronary intervention
TMAO: Trimethylamine N-oxide
STEMI: ST-elevation myocardial infarction
NSTEMI: Non ST-elevation myocardial infarction
