# Supplementary material for: Microbial signature of plaque and gut in acute coronary syndrome
Source: Sci Rep. 2023 Sep 7;13:14775. doi: 10.1038/s41598-023-41867-y (PMC10484905; doi:10.1038/s41598-023-41867-y)
Supplement: Supplementary file 1 — Supplementary Information. [file 41598_2023_41867_MOESM1_ESM.pdf]

Microbial signature of plaque and gut in acute coronary syndrome

Eugenia Pisano PhD[*ab], Francesca Bugli PhD[*cd], Anna Severino PhD[ab], Daniela Pedicino MD, PhD[a], Francesco Paroni Sterbini PhD[c], Cecilia Martini PhD[cd], Flavio De Maio PhD[c], Ramona Vinci, PhD[ab], Andrea Sacconi[e], Francesco Canonico PhD[ab], Alessia D'Aiello MD[ab], Alice Bonanni PhD[ab], Luca Proto[b], Pellegrino Ciampi MD[b], Myriana Ponzo MD[b], Maria Chiara Grimaldi MD[b], Andrea Urbani PhD[cd], Aniello Primiano[c] Jacopo Gervasoni PhD[c], Rocco Montone MD, PhD[a], Filippo Crea MD[ab], Maurizio Sanguinetti MD[cd§], Giovanna Liuzzo MD, PhD[ab§].

[a]Department of Cardiovascular Sciences, Fondazione Policlinico Universitario A. Gemelli IRCCS Rome, Italy.
[b]Department of Cardiovascular and Pulmonary Sciences, Catholic University of the Sacred Heart, Rome, Italy.
[c]Department of Laboratory and Infectious Sciences, Fondazione Policlinico Universitario A. Gemelli IRCCS, Rome, Italy.
[d]Department of Basic Biotechnological Sciences, Intensivological and Perioperative Clinics, Catholic University of the Sacred Heart, Rome, Italy.
[e]UOSD Clinical Trial Center, Biostatistics and Bioinformatics; Regina Elena National Cancer Institute- IRCCS-Rome, Italy.

Running Title: Gut and Plaque microbiotas in ACS and CCS patients

Funding: This work was supported RCR-2022-23682288 - Rete CARDIOLOGICA- Integrated strategies for the study of tissue and molecular determinants of vulnerable atherosclerotic plaque - Procedura nota DGRIC n. 1401 del 13/04/2022 Fondo progetti reti EF 2022 and PRIN 2017 Prot. 2017 WJBKKW_001.

Disclosures
All the authors have reported that they have no relationships relevant to the contents of this paper to disclose.

*Dr. Pisano and Dr. Bugli have contributed equally to this work as first authors
§Prof. Liuzzo, and Prof. Sanguinetti have contributed equally to this work as last authors
Corresponding author: Dr. Anna Severino
Department of Cardiovascular and Pulmonary Sciences, Catholic University of Sacred Heart
Largo F. Vito 1, 00168 Rome, Italy
Phone: +39 0630154187/6621
Email: anna.severino1@unicatt.it

Supplemental material

Supplemental Methods

1. Methods

1.1 Study populations

The exclusion criteria were as follows: 1) age >80 years; 2) acute cardiogenic shock and/or severe chronic heart failure (NYHA class III-IV, LVEF <35%); 3) severe heart valve disease; 4) recent (<3 months) major surgical procedures or trauma; 4) in-stent restenosis, stent thrombosis, or culprit lesion in a saphenous vein graft; 5) autoimmune diseases, evidence of immunologic disorders, acute or chronic infectious disease; 6) liver diseases; 7) use of anti-inflammatory or immunosuppressive drugs other than low-dose aspirin; 8) malignancies; 9) chronic kidney disease, stage 4 (GFR < 30 ml/min); and 10) treatment with antibiotics until 4 weeks prior.

1.2 Angioplasty balloon and stool sample collection

Angioplasty balloons were collected from 34 ACS and 11 CCS patients. A balloon predilatation was performed in all cases. Semicompliant balloons were chosen with a diameter of 0.5 mm less than the reference vessel diameter as assessed by visual estimation. After opening the sterile pack, the balloon was swiftly inserted into a Y connector (Copilot; Asahi Intecc/Abbott Vascular, Abbott Park, IL, USA) over the wire already positioned across the stenosis, taking care not to touch the balloon, and a 30 s dilatation was performed at nominal pressure. After balloon deflation, the balloon was removed from the Y connector by widely opening the valve to avoid entrapment of the balloon and loss of the material present on the balloon surface. The shaft was then cut a few cm from the proximal end of the balloon and placed into a sterile tube with a physiological solution. The plaque material was washed from the angioplasty balloons with sterile phosphate buffered saline under sterile conditions, centrifuged and then stored at -80 °C for sequencing analysis. Three balloons were used as negative controls; after opening the sterile pack, taking care not to touch the balloon, they were placed directly into a sterile tube with a physiological solution and then processed as the other samples. All steps were carried out under sterile conditions.

Stool samples were collected into Danastool Sample Collection microbiome tubes (Danagen) and stored at -80 °C. Stool samples from CCS, NSTEMI and STEMI patients were collected during hospitalization, after the PCI of the culprit lesion. At that time patients were in the sub-intensive care Unit. Stool samples from controls were collected at home and brought to the hospital on an outpatient visit.

1.3 Bacterial DNA Extraction

Bacterial DNA was extracted from stool samples using the QIAamp Fast DNA Stool Mini Kit (Qiagen, Hilden, Germany). DNA from the plaque material was extracted by a QIAsymphony (Qiagen, Hilden, Germany) automatic extractor following the manufacturer's instructions. DNA was quantitated with a Thermo Fisher Scientific NanoDrop ND-1000 spectrophotometer (Thermo Fisher Scientific, Waltham, USA). A negative control (molecular biology-grade pure water) was extracted in parallel and then processed together with the other samples. The three control balloons were extracted in parallel and then processed together with the other samples.

1.4 Evaluation of the presence of bacterial rDNA 16S in balloon samples by qualitative PCR

This initial analysis was performed on balloon samples to establish the presence of bacterial DNA. Each sample including the three control balloons were amplified for human β-globin and 16S bacterial rDNA (Supplementary Table 1). PCR was carried out with 0.3 μM forward and reverse primers with ≈20 ng template DNA and a 1X HotStar Taq Plus Master Mix kit (Qiagen, Hilden, Germany). Thermal cycling consisted of initial denaturation at 95 °C for 2 min followed by 40 cycles of denaturation at 95 °C for 20 s, annealing at 52 °C for 20 s, and extension at 65 °C for 60 s. Replicate amplicons were pooled, purified with an Agencourt AMPure Kit (Beckman Coulter, Brea, USA), and visualized by electrophoresis using 1.0% agarose gels. Only samples positive for β-globin and DNA 16S qualitative PCR were subsequently sequenced. Control balloons failed to produce visible PCR products and were not further analyzed.

1.7 Design of degenerate primers targeting the CutC and CntA genes

The degenerate primers used in ddPCR for the amplification of the CutC and CntA genes were built using a multiparametric approach. The GenBank database and Conserved Domain Database (CDD) at the National Center for Biotechnology Information (NCBI) were queried to select representative bacterial proteins of the choline trimethylamine-lyase protein family TIGR04394 (choline_CutC; EC Number 4.3.99.4) and the carnitine oxygenase protein family SSF50022 (CntA; EC Number 1.14.13.239), including the CutC enzymes of *Desulfovibrio desulfuricans* and *Klebsiella pneumoniae* and the CntA enzyme of *Escherichia coli*. The corresponding coding nucleotide sequences (CDS) of selected proteins were then used in the Basic Local Alignment Search Tool (BLAST) to find regions of local similarity between the CutC and CntA sequences. Partial gene sequences (<80% coverage) were omitted, and 100 sequences from CutC and CntA searches were used in Geneious Prime software for the design of the degenerate primers. Primers were designed in the highest homology regions of the consensus identified sequence. Applying a maximum degeneration percentage of 20%, the software produced three primer pairs for each gene. Each pair was tested by a qPCR efficiency curve using single genomic DNA from 8 bacterial strains and a mix of them (see Supplementary Table 1A). The degenerate primers with the highest efficiency level (>90%) were selected and used in further experiments. The selected primer sequences are depicted in Supplementary Table 1B.

1.8 Droplet digital PCR (ddPCR) of CutC and CntA genes

ddPCR (QX200 Droplet Digital PCR System, Bio-Rad Laboratories, Hercules, USA) was used to assess differences in CutC and CntA gene abundance between CCS, ACS and controls. We employed 16S rRNA abundances for normalization.

The reaction mixture consisted of 1x ddPCR Supermix for Evagreen (Bio-Rad Laboratories, Hercules, USA), 300 nM of each primer and 10 ng of DNA template. The samples were loaded into the droplet generator cartridge (Bio-Rad Laboratories, Hercules, USA), and 70 µl of droplet generation oil for Evagreen (Bio-Rad Laboratories, Hercules, USA) was added to the respective wells of the cartridge. The cartridge was covered with a gasket and placed in the QX200™ droplet generator (Bio-Rad Laboratories, Hercules, USA). The generated droplets were transferred to a 96-well PCR

plate, heat-sealed with a foil seal and placed into a C1000 TouchTM Thermal Cycler with a 96-Deep well Reaction Module (Bio-Rad Laboratories, Hercules, USA). Supplementary Table 2 lists the PCR cycling conditions. After completion of the PCR, the 96-well plate was inserted into the QX200™ droplet reader device (Bio-Rad Laboratories, Hercules, USA), and the fluorescence of each droplet was automatically counted.

Analysis of ddPCR data was performed using QX Manager 1.2. Droplets were clustered manually in 2D amplitude mode. The CutC and CntA abundances were calculated as the ratio of the fluorescence amplitude generated by CutC/CntA amplification and the fluorescence generated by rDNA16S.

1.9 Quantification of serum trimethylamine N-oxide levels

Trimethylamine-N-oxide (TMAO) was measured in plasma by ultraperformance liquid chromatography/mass spectrometry (UPLC-MS/MS). The UPLC-MS/MS system consisted of an UPLC Acquity (WATERS, Milford, MA, USA) and a triple quadrupole TQS-Micro (WATERS, Milford, MA, USA) equipped with an electrospray ion source. Analyses were performed in positive ion mode. The analytical procedure requires a deproteinization step, performed by adding 300 µL of acetonitrile containing of 2 µg/mL of [2H9] TMAO to 100 µL of plasma sample. After vigorous agitation, the sample was centrifuged at 10000 xg for 7 min at room temperature, and then the supernatant was transferred into a vial and injected into UPLC system for analysis.

Samples were loaded onto a LUNA HILIC column, 3 µm, 200 Å, 100 x 2.0 mm (Phenomenex, Torrance, USA). The chromatographic separation was performed with a gradient of mobile phase A (H_2O containing 5 mM ammonium acetate) and mobile phase B (acetonitrile containing 5 mM ammonium acetate), with a flow rate of 0.600 mL/min. The gradient followed this pattern: 0-2.0 min 99% B, 2.0-5.9 min 60% B, 5.9-6.0 min 30% B, 6.0-7.0 min 30% B, 7.0-7.1 min 99% B, 7.1-13.00 min 99% B. The oven temperature was set at 50 °C. The injection volume was 10 µL, and the total time analysis was 9.0 min. The optimized parameters for the ion source were: temperature at 400 °C, desolvatation gas at 1000 L/Hr, cone gas at 1 L/Hr, collision energy 30 V. Selected reaction monitoring was performed following the transitions m/z 75.9 à 58.4 for TMAO and 85.9 à 66.0 for

the internal standard [2H9] TMAO. Data acquisition was carried out using the mass spectrometer software (Waters MassLynx, Milford, USA).

Supplemental Figure Legend

Supplemental Figure 1. **Alpha-diversity analysis of the gut microbiota in patients with ACS, CCS and controls.** Shannon indices were used to evaluate the evenness and richness in the three groups.

Supplemental Figure 2. Beta-diversity analysis of the gut microbiota in patients with ACS, CCS and controls. (a) PCoA of the taxonomic abundance profiles based on the unweighted UniFrac distance between controls, ACS patients and CCS patients (F-value: 1.49; R2: 0.03; p< 0.02); (b) PCoA between controls and ACS patients (F-value: 1.66; R2: 0.02; p< 0.03), between controls and CCS (F-value: 1.75; R2: 0.03; p< 0.02), and between ACS and CCS (F-value: 1.01 R2: 0.01; p< 0.34). Differences in β- diversity were calculated with the PERMANOVA test.

Supplemental Figure 3. Correlation between deregulated taxa abundance and clinical features/risk factors/pharmacological therapies. Heatmap showing correlations of the deregulated taxa abundance with clinical features (a), risk factors (b) and pharmacological therapies (c). Spearman correlation coefficients are represented by color ranging from blue, negative correlation, to red, positive correlation; * $p < 0.05$.

Supplemental Figure 4. Beta-diversity analysis of the coronary plaque microbiota in patients with ACS and CCS. PCoA of the taxonomic abundance profiles, based on the unweighted UniFrac distance (PERMANOVA test). No significant difference was found between ACS and CCS patients.

Supplemental Figure 5. Overall comparison of the gut and coronary plaque microbiotas. Comparison of the microbiota alpha-diversity between gut and coronary plaque (p=1.2192e-16). Shannon indices were used to evaluate the evenness and richness in the two habits. Comparison of the microbiota β-diversity between gut and coronary plaque (F value: 79.102; R2: 0.41177; p=0.001).

Supplemental Figure 6. Microbial composition in the two habitats. Comparison of the mean relative abundances of bacterial phyla and genera in the coronary plaques (upper panels) and in the gut (bottom panels).

Supplemental Figure 7 CutC gene is significantly more abundant in CCS patients vs controls (p=0.0024), NSTEMI patients vs controls (p=0.0023) and STEMI patients vs controls (p=0.0082).

CntA gene is significantly more abundant in CCS patients vs controls (p=0.015) while STEMI patients vs controls and NSTEMI patients vs controls comparisons did not reach statistical significance.

Supplemental Figure 8. TMAO serum levels in patients with ACS and CCS. No significant difference was evident between ACS and CCS patients (a). Patients with STEMI showed an increased level of TMAO compared with NSTEMI and CCS, although the difference was not statistically significant (b).

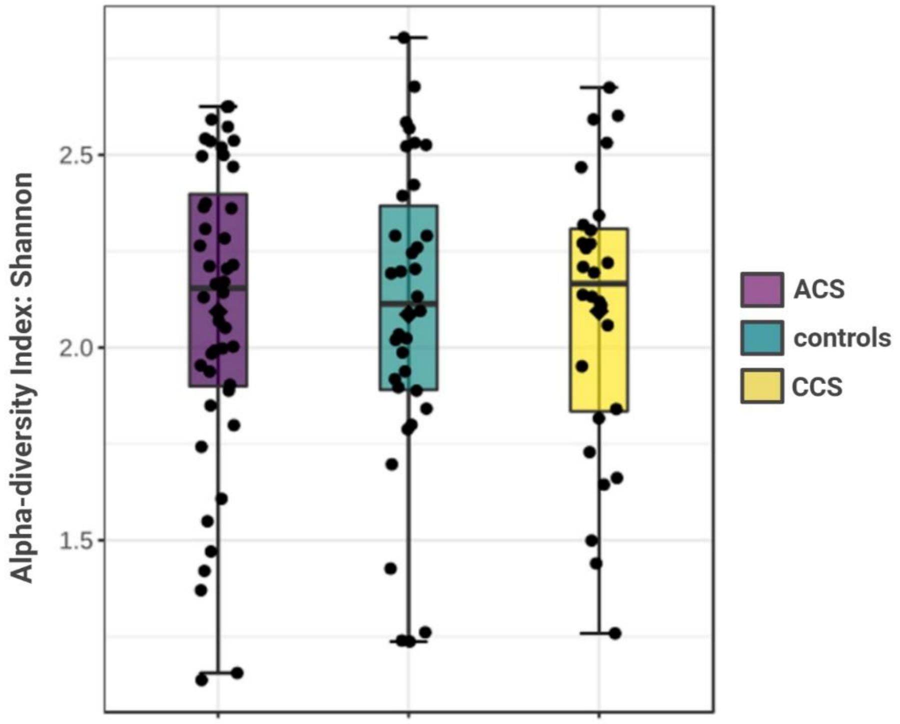

Figure S1

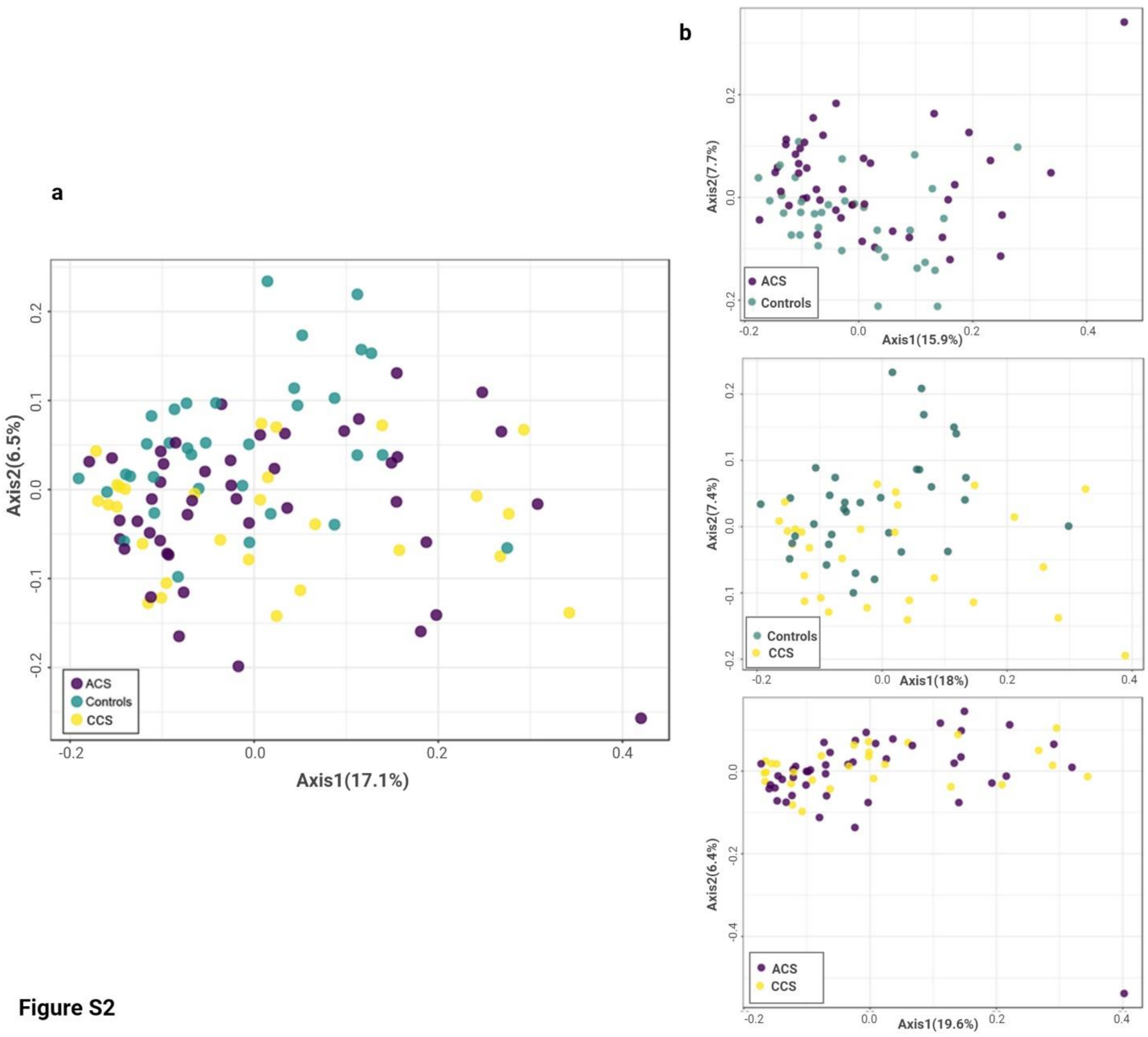
a
Axis2(6.5%)
Axis1(17.1%)
ACS
Controls
CCS
b
Axis2(7.7%)
Axis1(15.9%)
ACS
Controls
Axis2(7.4%)
Axis1(18%)
Controls
CCS
Axis2(6.4%)
Axis1(19.6%)
ACS
CCS

Figure S2

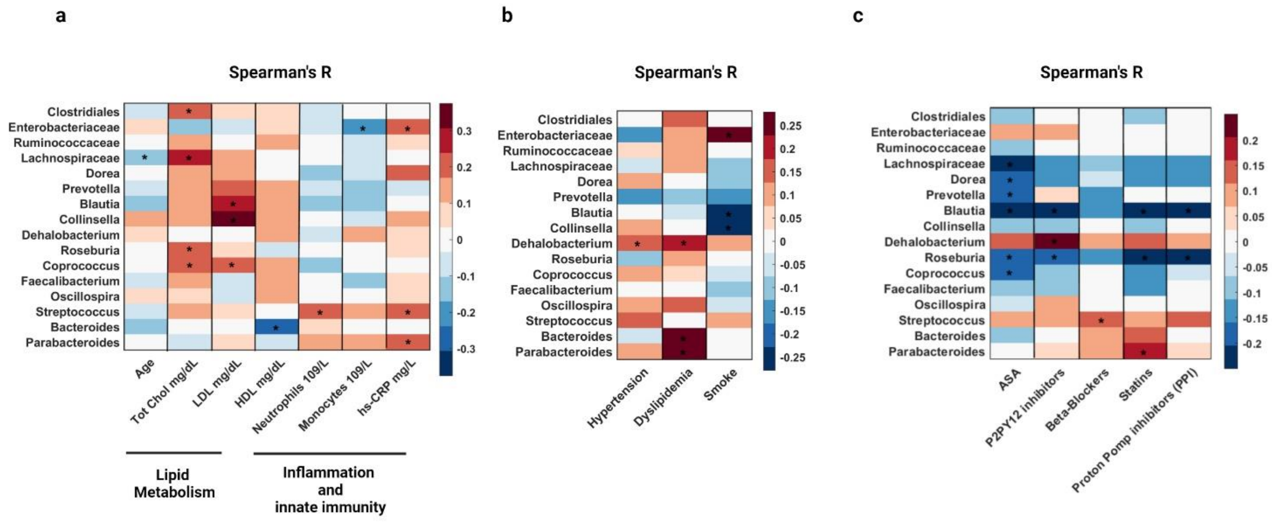

Figure S3

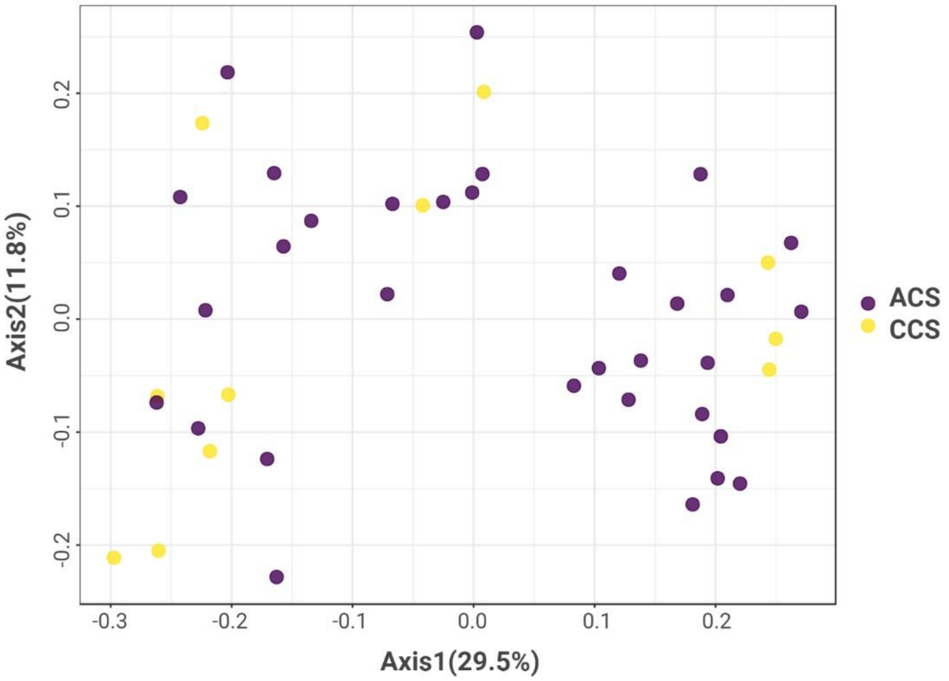

Figure S4

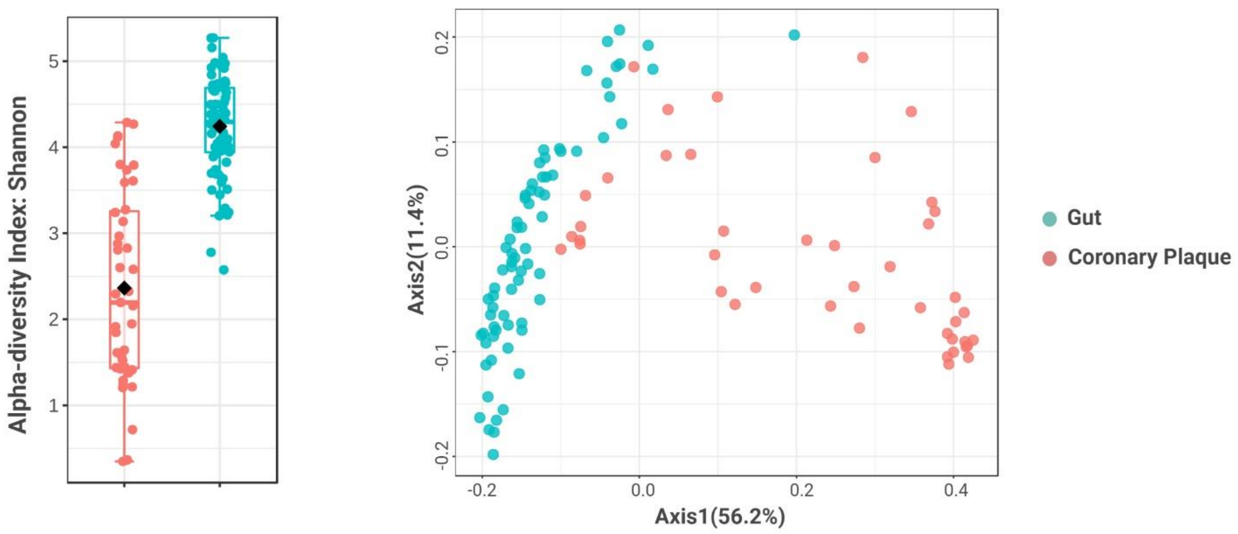

Figure S5

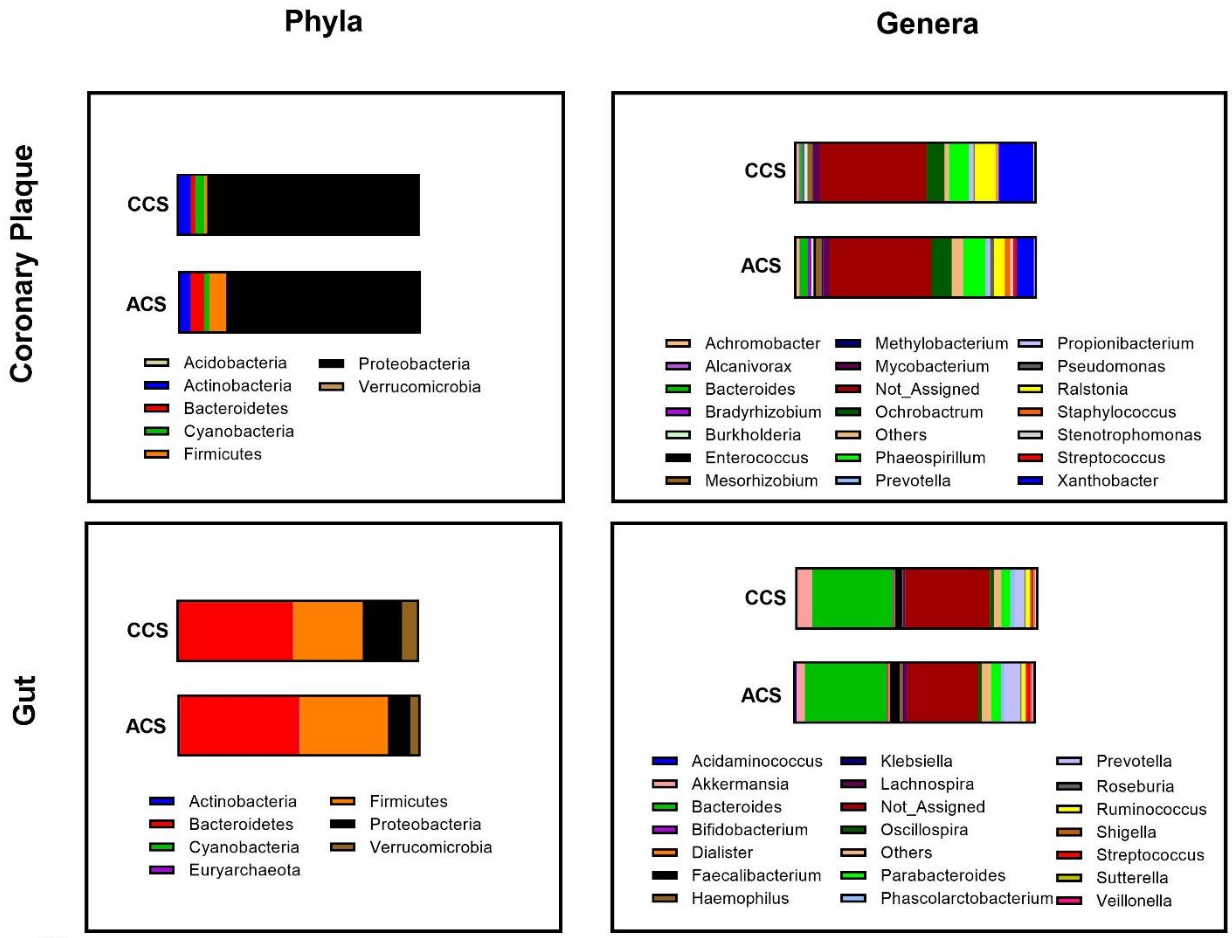

Figure S6

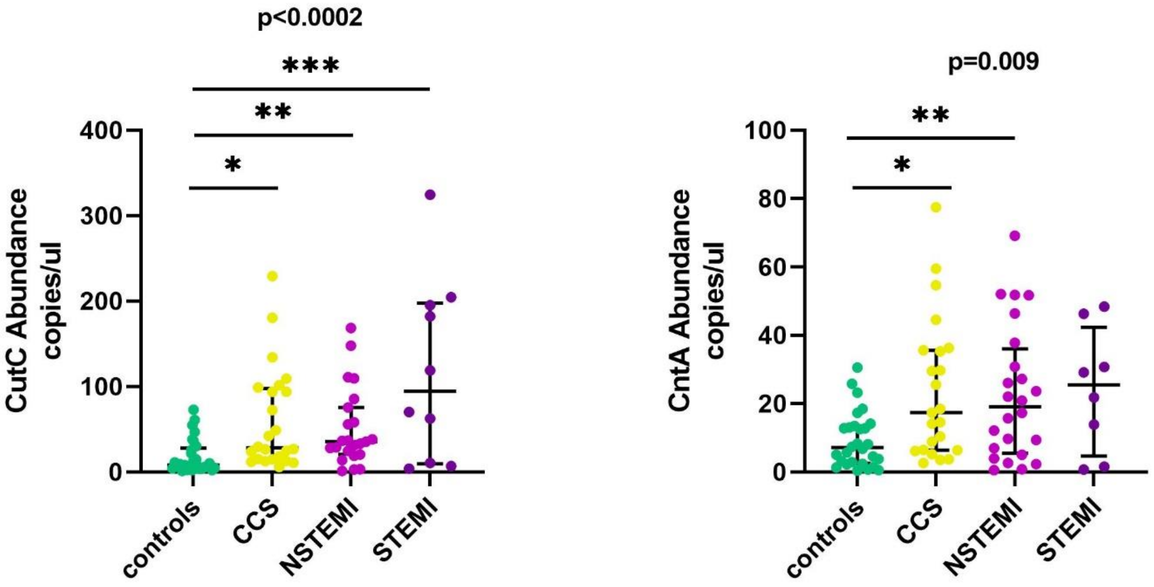

Figure S7

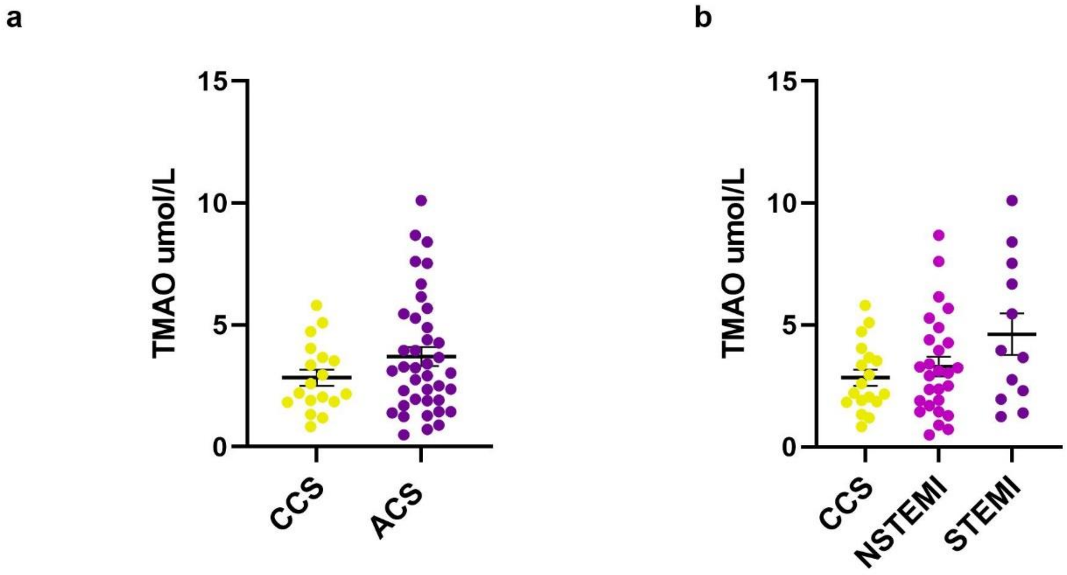

Figure S8

Supplemental Table 1a

List of bacterial strains

Genus	Species
Clostridium	difficilis
Streptococcus	dysgalactiae
Klebsiella	pneumoniae
Escherichia	coli
Aeromonas	hydrofila
Serratia	rubidiae
Providencia	stuarti
Proteus	mirabilis

Supplemental Table 1b

Primers used for qualitative PCR and ddPCR

Primer name	Primer Sequence 5' to3'
β-Globin forward	ACACAACTGTGTTCACTAGC
β-Globin reverse	GAAACCCAAGAGTCTTCTCT
16SrDNA Pro341F forward[22]	CCTACGGGNBGCASCAG
16SrDNA Pro805R reverse[22]	GACTACNVGGGTATCTAATCC
CutC deg forward	GCVGGHATGGGMTTCCCDGC
CutC deg reverse	TCNACRCARCCCATYARGCAGTA
CntA deg forward	CGGAABACRTYGCGRTACCA
CntA deg reverse	GGCCKTGCACCATGTTCAACG
